# Advances in the pathogenesis of *FLT3*-mutated acute myeloid leukemia and targeted treatments

**DOI:** 10.1097/CCO.0000000000001094

**Published:** 2024-08-26

**Authors:** Serena Travaglini, Carmelo Gurnari, Tiziana Ottone, Maria Teresa Voso

**Affiliations:** a Department of Biomedicine and Prevention, University of Tor Vergata; b Department of Experimental Medicine, University of Rome Tor Vergata, Rome, Italy; c Department of Translational Hematology and Oncology Research, Taussig Cancer Institute, Cleveland Clinic, Cleveland, Ohio, USA

**Keywords:** acute myeloid leukemia, FLT3 inhibitors, FLT3-ITD

## Abstract

**Purpose of review:**

*FLT3* mutations are among the most common myeloid drivers identified in adult acute myeloid leukemia (AML). Their identification is crucial for the precise risk assessment because of the strong prognostic significance of the most recurrent type of *FLT3* alterations, namely internal tandem duplications (ITDs). Recent advances in the pathogenesis and biology of *FLT3*-mutated AML have opened an opportunity for development and application of selective inhibition of FLT3 pathway.

**Recent findings:**

In the last decade, at least three targeted treatments have been approved by regulatory agencies and several others are currently under investigations. Here, we review the latest advance in the role of *FLT3* mutations in AML, providing an outline of the available therapeutic strategies, their mechanisms of actions and of resistance, as well as routes for potential improvement.

**Summary:**

The availability of FLT3 inhibitors has improved outcomes in AML harboring such mutations, currently also reflected in disease stratification and recommendations. Newer inhibitors are under investigations, and combinations with chemotherapy or other targeted treatments are being explored to further improve disease outcomes.

## INTRODUCTION

Acute myeloid leukemia (AML) with somatic alterations in the fms-like tyrosine kinase-3 (*FLT3*) gene represents approximately 25–30% of newly diagnosed adult AML cases [1^▪^,^2^]. This particular AML subtype is recognized as a separate nosological entity, and is associated with a poor prognosis due to a higher incidence of relapse [3,4].

In the past decade, several studies have thoroughly investigated the structure and function of FLT3 receptor, deeply highlighting the critical role of *FLT3* alterations as major driver events in the development of leukemia. Since their identification, activating mutations of *FLT3*, including internal tandem duplication (ITD) and point mutations in the tyrosine kinase domain (TKD), have been associated with a significant increase in the relapse rates, partly due to the persistence of *FLT3*-mutated subclones. These subclones are resistant to standard treatment and represent the seeds of subsequent disease relapse.

The evidence surrounding FLT3 biology strongly paved the way for its use as an attractive target for therapeutic intervention, which led to the development of novel treatment strategies, such as small-molecule inhibitors that directly target activated FLT3. However, data derived from clinical trials investigating the use of FLT3 inhibitors (FLT3i) as single agents showed a high variability of responses, making this subset of patients still at a high risk of disease recurrence [3,4]. This suggests the need to investigate novel therapeutic strategies that can improve the results obtained with FLT3i.

Herein, we will focus on *FLT3* molecular alterations and their potential impact on AML pathogenesis and prognosis, summarizing the design of treatments targeting FLT3, and discussing those currently under development. 

**Box 1:**
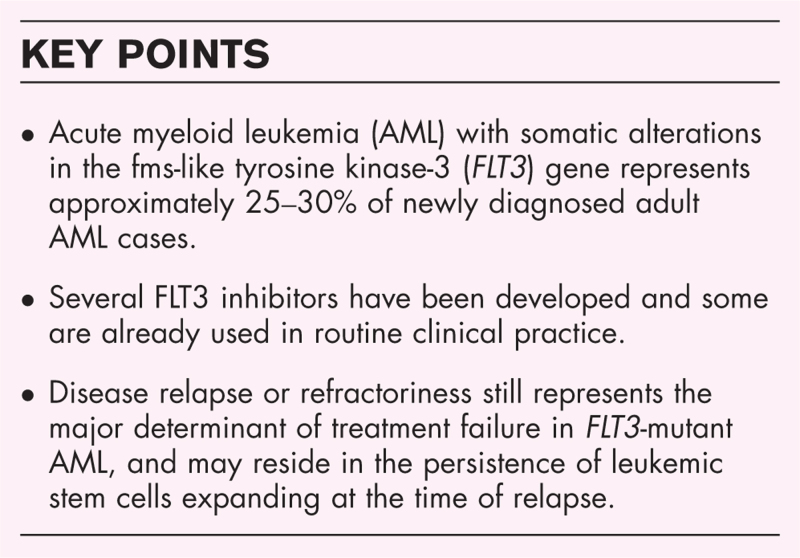
no caption available

## FLT3 IN ACUTE MYELOID LEUKEMIA PATHOGENESIS: MUTATIONS AND ONCOGENIC SIGNALING PATHWAYS

The *FLT3* gene on chromosome 13q12 encodes for a class III receptor tyrosine kinase (RTK), which plays a major role in the process of normal hematopoiesis, as well as in leukemogenesis.

The well defined modular structure of the FLT3 receptor is composed of distinct domains with specific and critical functions. The extracellular N-terminus domain is characterized by five immunoglobulin-like domains, being responsible for the binding of its ligand (FL). Furthermore, the compact transmembrane region facilitates the interaction among receptors, while the juxtamembrane region with a crucial regulatory domain by phosphorylation significantly impacts the activity of the TKD. The latter, in turn, is responsible for the enzymatic activity of the receptor. Upon interaction with FL, the receptor undergoes a conformational change, triggering homodimerization, autophosphorylation and activation of proliferative and pro-survival pathways, through RAS/MAPK, JAK/STAT5 and PI3K/AKT pathways [5,6].

Two common types of *FLT3* alterations have been considered clinically relevant, leading to the constitutive activation of FLT3 receptor: ITD mutations within the JM domain (exons 14 and 15), associated with an intermediate-risk feature [7^▪^], or point mutations in the TKD (exon 20), whose prognostic significance is still controversial [8]. The most frequently reported TKD mutation is a substitution of the amino acid at residue 835 with a tyrosine or histidine [9]. Apart from canonical *FLT3* mutations, rare point mutations, deletion or deletion/insertion alterations clustering in the JM domain of *FLT3*, have also been described in AML [10,11^▪▪^]. While these mutations exhibit clinical and biological features similar to *FLT3*-ITD variants, their precise role in the development of AML and the functional relevance especially in terms of response to currently available FLT3i still remains debated [12].

## PROGNOSTIC SIGNIFICANCE AND CLINICAL IMPLICATION OF *FLT3* GENE MUTATIONS ACCORDING TO THE LATEST DISEASE CLASSIFICATIONS

In the last 5 years, newly acquired biological and clinical data significantly contributed to the latest update of the ELN 2022 recommendations for AML diagnosis and treatment [2,13]. Accordingly, *FLT3*-ITD mutant AML is now included in the intermediate-risk category, regardless of the allelic ratio, studied by capillary electrophoresis. This has been the result of the availability of targeted treatment (see below) for *FLT3*-mutant AML, which in the previous ELN 2017 recommendations could be classified in any of the three-tiers classification system according to both *NPM1* mutational status and allelic ratio [14].

The current availability of targeted treatments has been a major advance and is reflected by better survival in this patient setting, prompting the above-mentioned update in the latest disease classifications. While ELN 2022 recommendations do not identify a threshold for allelic ratio by capillary electrophoresis as *FLT3*-ITD positivity, several laboratories still use the 0.05 cut-off established in the RATIFY trial, while others refer to next-generation sequencing (NGS) panels, not only for diagnosis but also for disease monitoring [15], as NGS or high-coverage PCR may enable detection of *FLT3*-ITD with 100 to 1000-fold greater sensitivity if compared to capillary electrophoresis [16,17]. Furthermore, some studies have also implied that not only *FLT3*-ITD clonal burden in terms of allelic ratio but also the length of the ITD might impact survival outcomes [18].

## THERAPEUTIC TARGETING OF FLT3

### FLT3 inhibitors: classification and mechanism of action

Since their development, the use of FLT3i as single-agent or in combination therapy has definitely improved patient outcomes, potentially allowing for less aggressive therapeutic regimens and, in some cases, even avoiding the need for cytotoxic chemotherapy [3].

FLT3i can be categorized in more than one way, based on the phase of drug discovery, specificity for the FLT3 receptor and mechanism of action. Concerning the phase of FLT3i discovery, first-generation inhibitors include midostaurin, sorafenib, sunitinib, lestaurtinib and tandutinib, all showing a low grade of specificity for FLT3, and also efficacy towards its downstream targets. More specific and potent are second-generation FLT3i [19], including gilteritinib, quizartinib and crenolanib, which displayed fewer toxicities and off-target effects. These inhibitors show a more potent activity against FLT3, sparing downstream targets [19]. Third-generation FLT3i, which include more effective molecules, have been designed to overcome resistance mechanisms and further improve clinical outcome for *FLT3*-mutated AMLs, and are currently under investigation [20].

According to their specificity for FLT3 and its downstream targets [21], FLT3i can be categorized into two types. Type I inhibitors (sunitinib, sorafenib, midostaurin, lestaurtinib), all exhibiting a broad activity beyond kinases, targeting other significant signaling molecules, like KIT, PDGFR, VEGFR, RAS/RAF and JAK2. In contrast, type II inhibitors (quizartinib, crenolanib, gilteritinib) offer enhanced specificity and potency for FLT3, with reduced off-target side effects.

**FIGURE 1:**
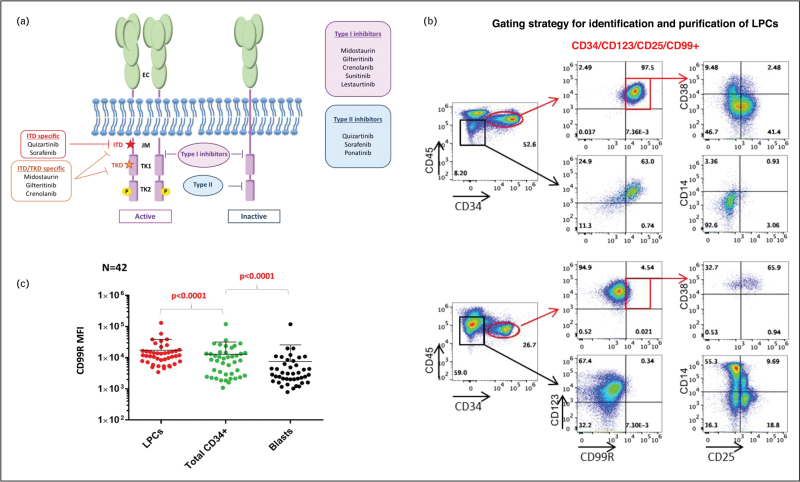
Targeting FLT3 in acute myeloid leukemia. A) Schematic representation of FLT3 receptor and inhibitors targeting specific domains. B) Gating strategy for leukemia progenitors cells. C) CD99 Mean Fluorescence Intensity in sorted cell populations from bone marrow samples of patients with FLT3 mutant AML.

A third classification is based on the mechanism of action of FLT3i. Type I inhibitors (sunitinib, lestaurtinib, midostaurin, crenolanib and gilteritinib), whose inhibitory activity relies on the binding to the ATP-binding site of the TKD, interact with both active and inactive states of FLT3 receptor, making them effective against both ITD and TKD mutations. Type II FLT3i (sorafenib, quizartinib, ponatinib) are instead specific for the inactive form of the receptor, binding to a pocket near the ATP-binding domain [19]. The structure of FLT3 receptor and the site of interaction of different FLT3i are illustrated in Fig. 1.

### FLT3i regimens and clinical implications

Several tyrosine kinase inhibitors have been studied in clinical trials for *FLT3*-mutated AML treatment. To date, only three FLT3i have received the approval from the Food and Drug Administration (FDA), European Medicines Agency (EMA) and Italian Medicines Agency (AIFA), including Midostaurin, Gilteritinib and Quizartinib, each used in distinct treatment settings.

For many years, intensive chemotherapy with ‘7+3’ regimen (3 days of daunorubicin and 7 days of cytarabine) has represented the standard treatment for *FLT3*-mutated AML. However, this paradigm shifted with the development of midostaurin, a multitargeted inhibitor that primarily inhibits FLT3 regardless of its subtype, redefining the treatment landscape for *FLT3*-mutated AML. Following the results of the phase III RATIFY trial, midostaurin in combination with standard 7+3 induction and consolidation chemotherapy (high-dose cytarabine), significantly improved overall survival (OS) and event-free survival (EFS) compared to chemotherapy alone, leading to the definition of this combination as the gold standard for newly diagnosed *FLT3*-mutated AML [22]. Interestingly, the median OS of all 360 patients in the midostaurin group reached 74.7 months, whereas the placebo group achieved a median OS of 25.6 months (*P* =  0.009). The midostaurin arm also displayed improved EFS (8 vs. 3 months) and reduced cumulative incidence of relapse (CIR, *P* = 0.01) [23]. Interestingly, no significant differences in the rate of severe adverse events were reported in the two arms [22]. While midostaurin now represents the standard of care for newly diagnosed *FLT3*-mutated AMLs, its use did not demonstrate durable clinical benefit in the maintenance setting, as emerged from RATIFY and RADIUS trials [24]. Furthermore, due to the high median age of onset for AML (68 yeas), most patients might not be eligible for this effective therapy, which is an add-on to standard chemotherapy, only offered to fit patients [25].

Among first-generation FLT3i, sorafenib has been extensively used in cancer treatment due to its potent antitumoral activity [20], and also tested in de-novo *FLT3*-mutated AML in many clinical trials, in combination with intensive chemotherapy. The lack of efficacy in the treatment of newly diagnosed AML, the high incidence of adverse events and the availability of more powerful and effective treatment options for *FLT3*-mutated AML, did not allow the approval of sorafenib as frontline therapy. However, the efficacy of its use as maintenance after allogeneic hematopoietic cells transplantation (allo-HCT) has been demonstrated by two distinct phase II trials, SORAML and SORMAIN, in *FLT3*-mutated AML patients aged less than 60 years. Data from the SORAML revealed that sorafenib was generally well tolerated. Most common side effects (grade 3-4) reported in the trial were similar between sorafenib and placebo arms and most of them were manageable by dose reduction [26]. Conversely, the SORAML study investigated the effect of sorafenib during the induction phase and reported a significant increase in the incidence of side effects, leading to 30% of patients discontinuing treatment [27]. Notably, real-world data have recently confirmed the safety and efficacy of sorafenib for *FLT3*-mutated AML in the posttransplant setting [28].

Next-generation inhibitors were devised to have more specific and potent activity with less toxicities, and longer half-life [29]. Among the second-generation FLT3i, the randomized phase III clinical study QuANTUM-First studied safety and activity of quizartinib in combination with standard induction and consolidation chemotherapy, with or without allo-HCT. The study enrolled 539 patients with a *FLT3*-ITD mutated AML at diagnosis [30^▪▪^]. Interestingly, this clinical trial addressed a key concern of the RATIFY trial, which excluded patients due to age, enrolling patients up to 75 years and providing valuable data for a more realistic cohort of AML patients [25]. As of patient outcomes, this study clearly demonstrated a statistically significant improvement in OS in the quizartinib versus placebo arm, associated with a reduction in CIR, deeper responses with achievement of measurable residual disease (MRD) negativity, and an increase in the duration of complete response (CR). Furthermore, as reported in the phase III QuANTUM-R study, the efficacy of quizartinib was also demonstrated as monotherapy in the R/R setting with significant improvement of OS versus salvage chemotherapy [31].

Beyond quizartinib, the phase I-II CHRYSALIS trial investigated the tolerability and safety, as well as the antileukemic effects of gilteritinib in relapsed/refractory (R/R) FLT3-mutated AML cases [32]. This study allowed to define the potent and durable inhibitory activity of gilteritinib against *FLT3*-mutated AML, as compared to a patient cohort treated with standard chemotherapy. These data were confirmed by the phase III ADMIRAL trial testing the efficacy of gilteritinib in the same R/R AML setting. This study showed significant improvement of patient outcomes as compared to salvage chemotherapy, with extended median OS and EFS, and a higher CR/CRi (CR with incomplete hematologic recovery) rates in the gilteritinib arm as opposed to controls. Although these results depicted gilteritinib as an effective treatment option in such setting, the high relapse rates after initial response underscores the need for further investigations.

### FLT3i resistance mechanisms and novel therapeutic strategies against FLT3-mutated acute myeloid leukemia

While the introduction of FLT3i as the standard of care for *FLT3*-mutated AML has substantially changed the treatment landscape and improved clinical outcomes, the development of resistance has become one of the most challenging issues [33]. Several resistance mechanisms affecting the clinical response to FLT3i have been described, which include both genetic and nongenetic causes [34,35].

Concerning primary resistance, the most common reason lies in the presence of on-target mutations at the time of diagnosis, that limit the activity of FLT3i [36]. Among nongenetic contributions, overexpression of antiapoptotic proteins, such as Bcl2 and MCL-1, seems to substantially grant resistance to FLT3i providing the ability to elude apoptosis [37]. Moreover, bone marrow stromal cells may play a role as crucial resistance mediators through the release of FL that interferes with FLT3 inhibition [38].

As to secondary resistance, which refers to relapsed cases after initial response to FLT3i, a key role has been attributed to the presence of novel secondary *FLT3* alterations acquired during treatment [39], and off-target mutations in members of the FLT3 downstream signaling pathways [40–42]. Recent research also described distinct molecular patterns at diagnosis and relapse, with loss of *FLT3* mutations at the time of disease recurrence due to the selective pressure of FLT3i treatment [43], and the persistence of *FLT3*-mutated resistant clones [52]. Particularly, clonal selection mechanisms with treatment-emergent mutations (e.g., activating RAS/MAPK pathway signaling), have been identified as the most recurrent escape mechanisms from FLT3i activity [44]. Moreover, CYP3A4 produced by bone marrow stromal cells may degrade FLT3i [45].

The primary and secondary resistance mechanisms outlined previously can lead to the selection of leukemic stem cells (LSCs), which are essential for disease progression and relapse. In contrast to the bulk of leukemic cells, quiescent LSCs remain in a dormant phase of the cell cycle, which markedly decreases their sensitivity to standard chemotherapeutic agents designed to target rapidly dividing cells [46]. Understanding these resistance mechanisms is crucial for developing more effective treatments in the context of *FLT3*-ITD mutated AML. It is widely believed that LSCs are found within the CD34+/CD38- cell fraction. Research efforts have aimed to identify new therapeutic targets for AML, revealing that antigens like CD123 [47] and CD99 [48–50] are expressed on LSCs but not on normal HSCs. Through a multiparameter flow cytometry (MPFC) approach, Angelini *et al*. [51] demonstrated that the leukemia-associated CD34/CD123/CD25/CD99+ immunophenotype of the leukemic precursor cells (LPCs), is able to predict for *FLT3*-ITD mutations in AML patients with a specificity and sensitivity of more than 90%. The authors demonstrated that small FLT3-ITD mutated clones have clinical significance in AML, as they can appear following therapy in patients initially identified as *FLT3* wild-type. Such findings have been confirmed over the years by several groups [52,53], suggesting that one of the mechanisms of treatment failure may be the persistence of *FLT3*-ITD mutant LSCs at the time of complete morphological remission, acting as source of relapse [40]. To investigate the subclonal structure of *FLT3*-ITD mutant AML, our group explored the distribution of the *FLT3*-ITD mutation among different progenitor cell subsets in AML patients. We found that CD34/CD123/CD25/CD99+ LPCs had a significantly higher *FLT3*-ITD mutation load compared to CD34+ progenitors and mononuclear cells. Notably, this rise in allelic burden was even more significant when evaluated in the context of subclonal *FLT3*-ITD mutations, being also associated with a marked increase in CD99 mean fluorescence intensity. The CD99 antigen, a heavily O-glycosylated transmembrane protein, promotes the LSCs aggressiveness by enhancing transendothelial migration and mobilization of leukemia cells [49,50]. Notably, in-vitro experiments showed that an anti-CD99 mAb treatment was effective in eliminating CD34/CD99+ *FLT3*-ITD mutant LPCs resistant to midostaurin, with no impact on healthy CD34+ cells [49,54].

Numerous clinical trials are currently underway to investigate the use of FLT3i in combination with other targeted therapies, possibly identifying synergistic actions (Table 1). The efficacy of the first-generation inhibitor sorafenib has been tested in combination with azacitidine in both newly diagnosed and R/R *FLT3*-ITD mutated AML in two distinct phase II trials [55]. Moreover, the more potent second-generation FLT3i gilteritinib showed promising results in combination with azacitidine in both frontline and R/R settings [56]. However, these findings were not confirmed by the randomized phase III LACEWING trial (ClinicalTrials.gov: NCT02752035) in the older/unfit *FLT3*-mutated AML, which did not show significant differences in OS between the gilteritinib plus azacitidine versus the azacitidine monotherapy arm.

**Table 1:** FLT3 inhibitors in currently ongoing clinical trials for AML

FLT3i	Target	NCT	Target population	Clinical trials description	Protocol phase	Primary endpoint
Midostaurin	ITD, TKD	03836209	Previously untreated *FLT3* mutated Non M3 AML (FLT3-TKD or FLT3-ITD)	Gilteritinib vs Midostaurin in *FLT3* mutated Acute Myeloid Leukemia	2	FLT3 Mutation Negative Composite Complete Response (CRc) [includes Complete Response (CR) or CR with incomplete hematologic recovery (CRi)] at the end of Induction
	ITD, TKD	04174612	Previously untreated *FLT3* mutated (*FLT3*-TKD or *FLT3*-ITD)	“3+7” plus Midostaurin in *FLT3* mutated Acute Myeloid Leukemia	3	Event-Free Survival at 2.5 years
	ITD, c-KIT	01830361	Previously untreated c-KIT mutated t(8;21) AML with c-*KIT* and/or *FLT3*-ITD mutations	Midostaurin plus standard primary chemotherapy in c-*KIT* or *FLT3*-ITD mutated t(8;21) AML	2	Event-Free Survival at 2 years
	ITD, TKD	02723435	Elderly patients with *FLT3*-ITD/TKD post allogeneic hematopoietic cell transplant (HCT)	Midostaurin as maintenance therapy for elderly AML patients with *FLT3* mutations	2	Overall Survival at 1 year
Quizartinib	ITD	04676243	Previously untreated *FLT3-*ITD mutated AML	Quizartinib plus SOC chemotherapy vs treatment according to physician's choice in patients with *FLT3*-ITD AML	3	Modified Event-Free Survival (mEFS) as time from randomization to failure to achieve CR/CRi/CRh, relapse, or death, up to 4 years
		03793478	Previously untreated *FLT3-*ITD mutated AML in pediatric patients aged ≥1 month to ≤21years	Quizartinib in combination with re-induction chemotherapy and as a single-agent continuation therapy in pediatric patients with relapsed/refractory AML	1/2	Composite Complete Remission (CRc) rate after up to 2 re-induction cycles
		04209725	Relapsed/refractory *FLT3*-ITD AML previously treated with standard induction chemotherapy with or without FLT3 inhibitors	Quizartinib in combination with CPX-351 to treat relapsed or refractory *FLT3*-ITD mutated AML	2	Overall response rate [includes CR or CRi in patients receiving CPX-351 and Quizartinib] measured from cycle 1 day 1 until disease progression, up to 2 years
Sorafenib	ITD	04788420	*FLT3*-ITD AML who are recipients of allogeneic hematopoietic cell transplantation (allo-HCT)	Sorafenib maintenance after allo-HCT for *FLT3*-ITD mutated AML	2	Incidence of leukemia relapse at 2 years
		03622541	Adult AML patients with *FLT3*-ITD and persistent leukemia after at least two prior chemotherapy regimens	Sorafenib as salvage treatment for relapsed/refractory AML with *FLT3*-ITD	2	Evaluating Treatment Efficacy
Gilteritinib	ITD, TKD	02014558	Relapsed or refractory AML adult patients who have failed previous treatments or relapsed after remission	Gilteritinib in relapsed or treatment-refractory AML	1/2	Safety and tolerability
		NCT04240002	Relapsed or refractory AML pediatric patients aged ≥6 months to ≤21years	Gilteritinib with standard chemotherapy in Children, Adolescents and Young Adults with *FLT3*-ITD Relapsed/Refractory AML	1/2	Safety and tolerability during the first 28 days of phase 1, and the rate of CR and CRc after up to 56 days of therapy in phase 2
Crenolanib	ITD, TKD	NCT02283177	Newly diagnosed *FLT3* mutated Non M3 AML (*FLT3*-TKD or *FLT3*-ITD)	Crenolanib in combination with chemotherapy in *de novo* AML with *FLT3* mutations	2	Response rate to crenolanib with standard induction chemotherapy, including CR, CRi and partial response over 2 years
		NCT03250338	Adult AML carrying *FLT3* mutations, refractory or relapsed after first-line or second-line treatment	Crenolanib plus Chemotherapy vs chemotherapy alone in relapsed/refractory *FLT3* mutated AML	3	Event-Free Survival at 3 years
		NCT02400255	*FLT3* mutated AML who are recipients of allogeneic hematopoietic cell transplantation (allo-HCT)	Crenolanib maintenance after allo-HCT for *FLT3* mutated AML	2	Number of patients who relapsed during or after crenolanib maintenance therapy

Another route to overcome disease refractoriness has been the combination of FLT3i with the Bcl2 inhibitor Venetoclax. In fact, the upregulation of antiapoptotic proteins represents one of the previously mentioned mechanisms of resistance to FLT3i. Interestingly, the use of gilteritinib in combination with venetoclax in R/R FLT3-mutated AML showed a higher CR rate as compared to single-agent gilteritinib, as demonstrated by the ADMIRAL trial [57].

The observation of such results obtained with venetoclax prompted the testing of this drug in combination with the hypomethylating agent azacitidine and gilteritinib in both newly diagnosed AML patients and relapse/refractory cases unfit for intensive chemotherapy [58^▪^]. This strategy led to impressive rates of CR/Cri in the naive setting, deep FLT3 molecular responses and encouraging survival in the refractory cases. Furthermore, novel small molecule inhibitors are under currently investigation in both in-vitro (e.g., CD99) and in-vivo studies.

## CONCLUSION

*FLT3*-ITD mutations identify one of the most common subtypes of adult AML for which different targeted treatments have been made available in the last decade. Translational research studies have clarified mechanisms underlining treatment resistance, and opened routes for generations of novel FLT3i, which are currently under investigation in several clinical trials.

## Acknowledgements

*None.*

### Financial support and sponsorship

*This work was supported by AIRC 5x1000 call “Metastatic disease; the key unmet need in oncology” to MYNERVA project, #21267 (Myeloid Neoplasms Research Venture AIRC. A detailed description of the MYNERVA project is available at* *http://www.progettoagimm.it* *); PRIN grant No. 2017WXR7ZT; PRIN grant No. 2022KRA3JF; PRIN grant No P2022W25EA; Ministero della Salute, Rome, Italy (Finalizzata 2018, NET 2018 12365935, Personalized medicine program on myeloid neoplasms: characterization of the patient's genome for clinical decision making and systematic collection of real-world data to improve quality of healthcare) to M.T.V., MUR-PNRR M4C2I1.3 PE6 project PE00000019 Heal Italia to M.T.V. and S.T.; C.G. was supported by a grant from the Edward P. Evans Foundation.*

### Conflicts of interest

*The authors declare no competing financial interests.*

## REFERENCES AND RECOMMENDED READING

Papers of particular interest, published within the annual period of review, have been highlighted as:

▪ of special interest
▪▪ of outstanding interest
